# Microfacies analysis and diagenetic history of Lower to Middle Eocene carbonates at Umm Russies area in the northeastern desert of Egypt

**DOI:** 10.1038/s41598-025-05365-7

**Published:** 2025-06-20

**Authors:** Mahmoud Abd-Elhameed, Gamal Attia, Yasser Salama, Asmaa El-Moghazy, Abdelaziz Mahmoud

**Affiliations:** 1 Geology Department, Faculty of Science, Helwan University, Helwan, Egypt; 2 Geology Department, Faculty of Science, Beni-Suef University, Beni Suef, Egypt

**Keywords:** Lower-Middle eocene, North Eastern desert, Microfacies analysis, Diagenetic history, Paragenetic model, Geology, Petrology, Sedimentology

## Abstract

An integrated study, incorporating field observations and petrographic analysis, has been conducted on the Lower-Middle Eocene carbonates of the Umm Russies area, North Eastern Desert, Egypt. These carbonate sequence represented, from base to top, by Minia, Gebel Hof, and Observatory formations, primarily consisting of marl, dolomite, and limestone. The microfacies analysis allowed the identification of seven distinct microfacies types: bioclastic floatstone, ferruginous dolomite, bioclastic packstone, ferruginous peloidal grainstone, sandy rudstone, foraminiferal wackestone, and bioclastic packstone. These microfacies types reflect a deposition in a wide range of environments from high-energy inner ramp to low-energy middle ramp settings. The performed petrographic analysis indicates that the investigated carbonate rocks underwent significant modification through a range of diagenetic processes, such as micritization, glauconitization, and dolomitization, representing marine-phreatic, meteoric-phreatic, burial, and meteoric-vadose environments. These environments are part of three successive diagenetic stages; eogenesis, mesogenesis, and telogenesis. The relationship between diagenetic episodes and depositional settings highlights that high-energy inner ramp environments facilitated early cementation and micritization, while middle ramp conditions promoted dissolution and neomorphism. Restricted platform margin environments favored dolomitization and glauconitization. Integrating microfacies analysis with these diagenetic interpretations facilitates reconstructing paleoenvironmental conditions and provides a framework for understanding carbonate rock formation in various geological settings.

## Introduction

Carbonate ramps, ranging from inner to outer ramps, are prevalent along the continental margin of Tethys and are dominated by larger benthic foraminifera, including nummulitids and alveolinids, during the Eocene^1–3^. Investigation of these foraminifera provides essential insights into the paleoenvironmental conditions of Eocene carbonates^4–6^. During the Early Eocene, larger benthic foraminifera such as *Nummulites*, *Alveolina*, and *Assilina* were dominant along the Tethys margins, thriving in warm, shallow marine environments and contributing significantly to carbonate sedimentation^7^.

In the Middle Eocene, genera like *Nummulites*, *Assilina*, and *Asterocyclina* continued to dominate, reflecting persistent favorable conditions for carbonate platform development and providing critical insights into paleoenvironmental settings^8^. In Egypt, a well-defined Eocene sequence extends across the region from east Cairo in an east-west direction, with Eocene rocks exhibiting diverse lithologies and depositional settings^9^. The lithological complexity and tectonic influences associated with the opening of the Gulf of Suez have significantly impacted the depositional architecture of these areas^10,11^.

The Lower-Middle Eocene successions in northern Egypt are characterized by shallow marine ramp carbonates, with different marine facies (e.g. limestone, shale, marl, and chalk) and various faunal elements (e.g. corals, bryozoans, and benthic larger foraminifera)^12–14^.

**Fig. 1:**
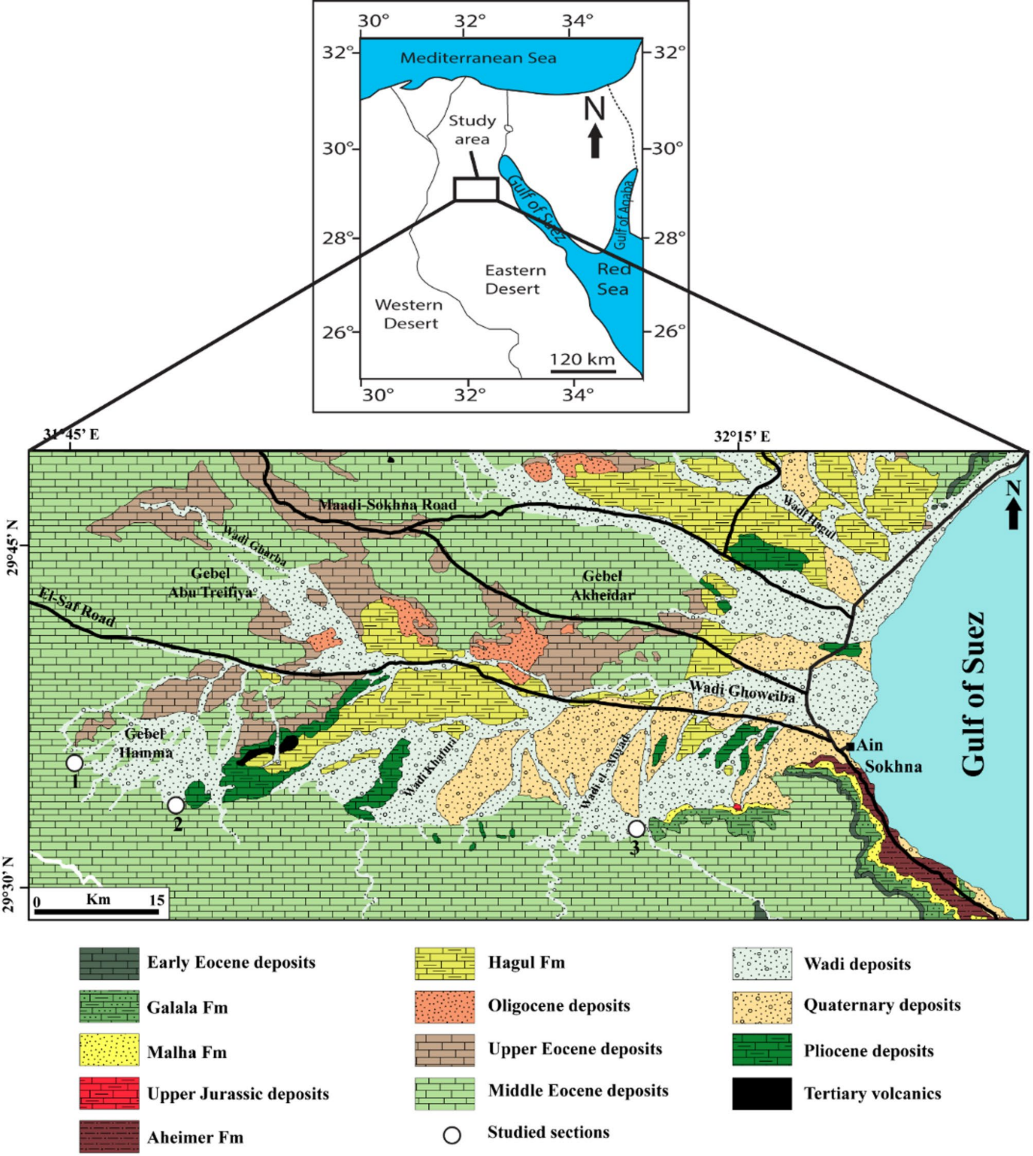
Location and geologic maps of the study area showing the studied Sect. 1: El-Hamma section, 2: Wadi El-Qanaa section, 3: El-Moniedra section, modified after Conoco^15^. The map was generated using QGIS (version 3.28), an open-source software for geographic data analysis.

This study investigates dispatched outcrops of the Lower-Middle Eocene ramp carbonates of Umm Russies area, north Eastern Desert, Egypt (Fig. 1). This area is a part of the Eocene Plateau that extends from Luxor to Cairo, representing the southern margin of the former Neo-Tethys Ocean. Despite the extensive literature on the Lower-Middle Eocene outcrops in north Eastern Desert from stratigraphical, paleontological and sedimentological points of view [e.g.;^5,9,12,16–19^], only the work of El-Dawoody and Galal^16^ was reported from the study area. El-Dawoody and Galal^16^ provided a micropaleontological analysis of the exposed Lower-Middle Eocene deposits, overlooking the detailed characteristics of the microfacies types and the diagenetic features of these deposits.

Therefore, the objectives of the present study are to discuss the stratigraphy of the Lower-Middle Eocene successions at El-Hamma, Wadi El-Qanaa, and El-Moniedra areas, Umm Russies area, north Eastern Desert, Egypt, to describe and interpret the different microfacies types of the studied successions, to propose a depositional model, to describe the various diagenetic processes affecting the investigated successions and their paragenetic sequence, and to propose a diagenetic model for these successions.

## Geological setting

The Eocene facies evolution in Egypt was influenced by global eustatic sea-level changes, synsedimentary tectonics, basin relief, terrigenous supply, and climatic conditions^20^. During the Eocene time, Egypt experienced significant tectonic events, including Syrian Arc folding and the Alpine orogeny^21^. Syrian Arc tectonism induced slope instability and remobilization of platform carbonate deposits in the northern Eastern Desert and Sinai^22^. In contrast, a stable shelf area south of Cairo, characterized by a thick accumulation of Cretaceous to Eocene marine carbonates and shales with minimal lithological variations, indicates less influence from Syrian Arc tectonics^22^.

At the end of the Middle Eocene, a regional unconformity developed due to the primary compressional event between the African and European plates^23^. Lotfy and Van der Voo^24^ revealed that Egypt’s paleogeographic latitude during the Middle-Late Eocene (15º-17º N) reflects predominant tropical climatic conditions.

During the Eocene time, extensive carbonate platform developed across a broad region of Egypt^25–27^. These carbonate deposits are occasionally associated with varying amounts of siliciclastic material^28^.

**Fig. 2:**
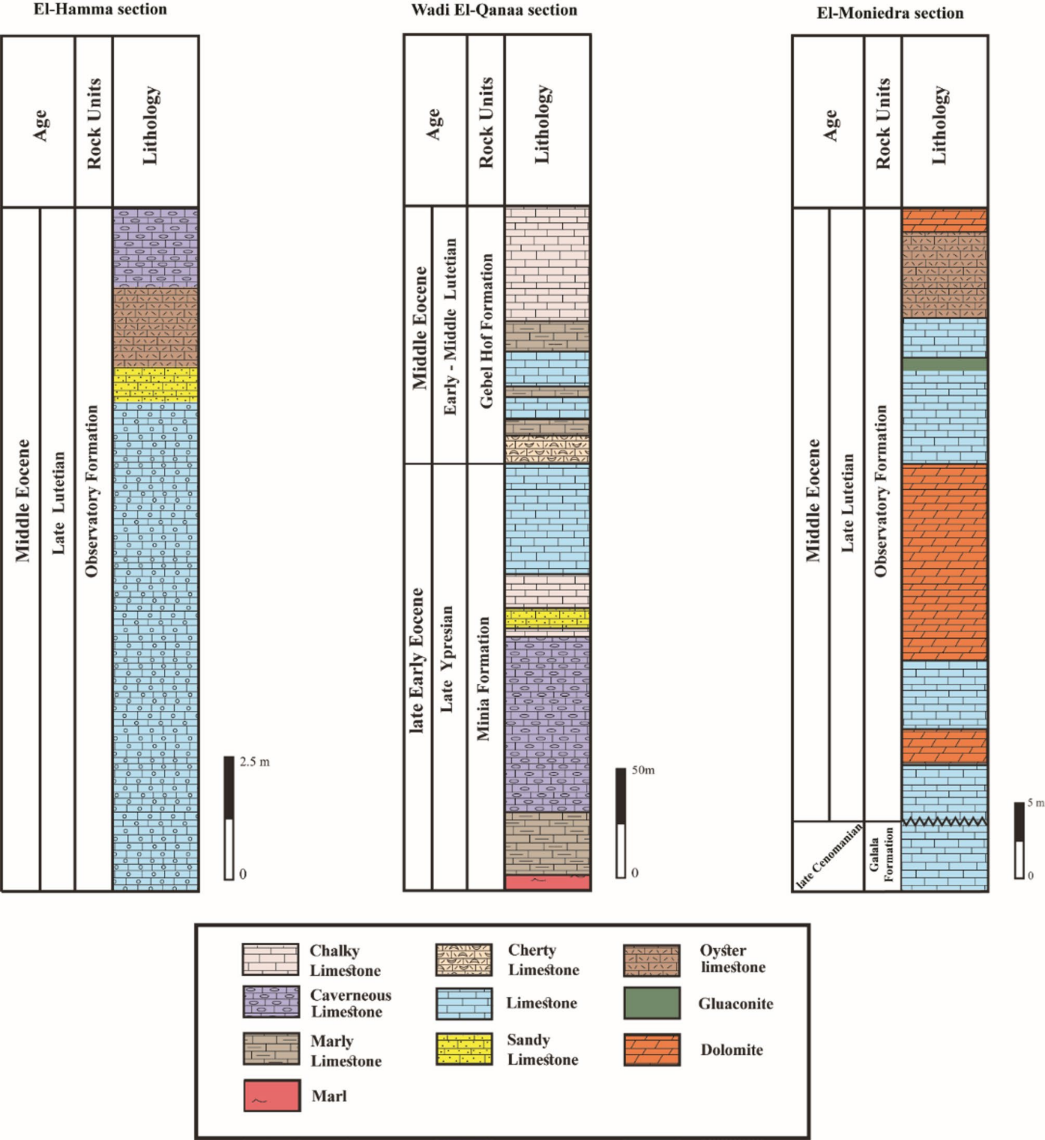
Lithostratigraphic sections at El-Hamma, Wadi El-Qanaa, and El-Moniedra areas.

The Eocene deposits represent approximately 21% of Egypt’s surface area, with several thousand meters thick. In the study area, the Eocene deposits are represented by late Early Eocene and Middle Eocene rocks. These deposits are defined by three distinct rock units, from base to top are; Minia, Gebel Hof, and Observatory formations (Fig. 2).

Additionally, volcanic sheets, primarily basalts, associated with the reactivation of E–W and NW–SE normal faults during the Late Oligocene and Early Miocene, have been reported in the study area. These basalts are dated to the Late Oligocene-Early Miocene [Aquitanian; 22 ± 2 Ma;^29^].

## Materials and methods

The present study integrates stratigraphic, petrographic, and microfacies analyses to interpret the depositional settings and diagenetic history of the Lower-Middle Eocene carbonate successions at El-Hamma, Wadi El-Qanaa, and El-Moniedra areas, Umm Russies area, North Eastern Desert, Egypt. Three stratigraphic sections were systematically measured and described, documenting lithological variations, sedimentary structures, grain composition, and fossil content (Fig. 1). Representative samples were collected on a bed-by-bed basis to ensure detailed laboratory analyses, forming the foundation for interpreting the depositional environments and diagenetic processes.

A total of 56 thin sections were prepared from the collected carbonate rock samples and examined under a polarizing microscope. This analysis focused on identifying microfacies types and documenting diagenetic features. The classification of microfacies followed the schemes of Dunham^30^ and Embry and Klovan^31^. Microfacies types were interpreted with respect to depositional environments by comparing them with the Standard Microfacies Types (SMF) of Flügel^32^ and the Facies Zones (FZ) of Wilson^33^. These interpretations highlighted the transitions between inner and middle ramp depositional settings.

Diagenetic features, including micritization, glauconitization, cementation, dolomitization, neomorphism, dissolution, silicification, compaction, fracturing, and iron oxides, were described and interpreted to reconstruct the diagenetic history (Table 1). These processes were integrated into a paragenetic sequence, providing insights into the diagenetic stages of eogenesis, mesogenesis, and telogenesis.

**Table 1:** Diagenetic features and interpretations of the Lower-Middle Eocene carbonates.

Diagenetic feature	Description	Interpretation
Micritization	Skeletal particles exhibit micrite envelopes, often with microborings; thickness 18–46 μm	Occurs in marine-phreatic environments under tranquil water conditions; stabilizes carbonate grains and prevents further diagenetic alteration
Glauconitization	Dispersed yellowish to reddish-green glauconitic grains; partial replacement of biogenic materials	Indicates sub-oxic, mild reducing conditions; formed through glauconitization of fecal pellets or biogenic materials in restricted marine environments
Cementation	Includes fibrous calcite, granular spar, and blocky calcite cements	Fibrous calcite formed in marine-phreatic settings; granular and blocky spar precipitated in meteoric-phreatic and burial diagenetic environments
Dolomitization	Two modes: matrix-replacive and void-filling dolomites; euhedral to subhedral crystals	Early dolomitization occurred in marine-phreatic environments; void-filling dolomites formed during shallow burial diagenesis
Neomorphism	Micritic matrix recrystallized to neomorphic spar; skeletal allochems partially replaced	Indicates stabilization of metastable aragonite and high Mg-calcite in meteoric-phreatic diagenetic conditions
Silicification	Silica occurs as pore-filling material and partial replacement of skeletal grains	Derived from terrestrial sources; precipitated during regression periods under meteoric-phreatic diagenesis
Compaction	Mechanical compaction caused grain deformation, fracturing, and reduced porosity	Reflects burial diagenesis; initiated shortly after deposition and progressed during shallow burial
Fracturing	Macroscopic and microscopic fractures occur in all formations	Indicates late diagenetic processes, including tectonic stress during uplift and meteoric-vadose diagenesis
Dissolution	This affected matrix, cements, and skeletal grains, creating secondary porosity	Occurred during meteoric-phreatic and meteoric-vadose conditions; dissolution facilitated later cementation
Iron oxides	Occurs as brown to reddish-black patches, often coating allochemical constituents	Represents epidiagenetic processes in oxidizing meteoric-vadose conditions; linked to subaerial exposure and weathering

The sequence stratigraphy was determined by integrating lithostratigraphic observations with detailed microfacies analysis. Sequence boundaries were identified based on variations in sedimentary facies and sequences of depositional units, and these boundaries were correlated with the well-established regional sequence stratigraphy models. The sequence stratigraphic framework follows the criteria of Catuneanu^34^ and, Catuneanu et al.^35^ Maximum flooding surfaces (MFS) and sequence boundaries were used to construct the overall sequence stratigraphic framework for the study area, providing insights into sea-level fluctuations. The depositional and diagenetic models were then integrated to understand the interplay between sedimentation and post-depositional changes.

## Results

### Lithostratigraphy

The sequence stratigraphy in the study area is represented by three stratigraphic rock units, belonging to the late Early to Middle Eocene time. These rock units are, from older to younger, Minia Formation, Gebel Hof Formation, and Observatory Formation (Fig. 2). A detailed description of each rock unit in the study area is given below.

Minia Formation was first introduced by Said^36^ to describe a 35-meter-thick section of thick-bedded, white, alveolinid limestone containing *Alveolina frumentiformis* Schwager and *Orbitolites* cf. *complanatus* Lamarck at Zawiet Saada area, opposite to Minia City. In the study area, it is only recorded at Wadi El-Qanaa area, with a thickness of about 200 m, representing the oldest Eocene rock unit, unconformably overlying the Upper Cretaceous (Cenomanian) Galala Formation (Fig. 2).

**Fig. 3:**
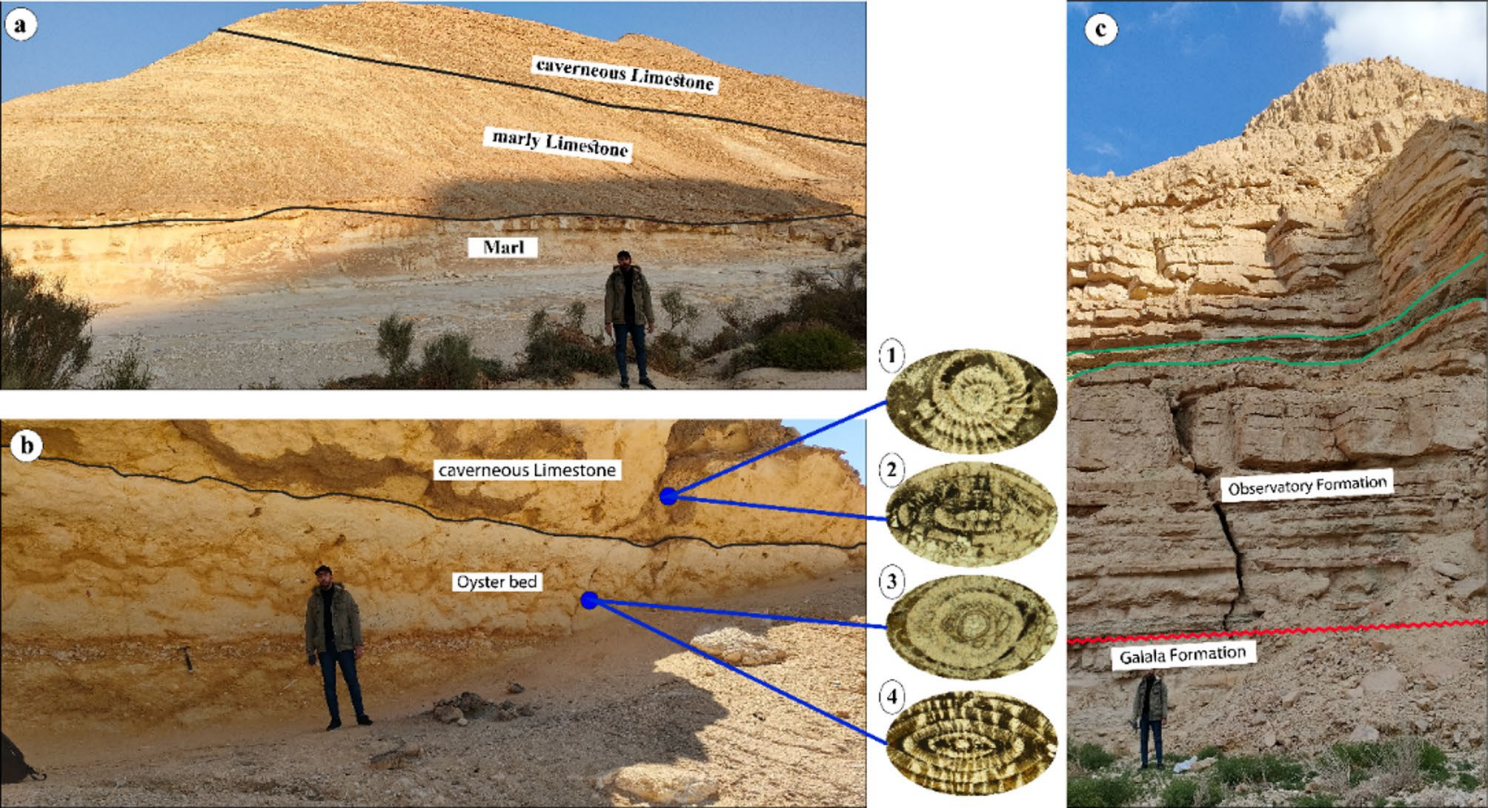
Field photographs of the studied lithostratigraphic units. (**a**) The lower part of Minia Formation at Wadi El-Qanaa area, marking the transition among the marl, marly limestone, and the overlying cavernous limestone. (**b**) The upper part of Observatory Formation at El-Hamma area, consisting of thick oyster bed followed by cavernous limestone. 1: *Nummulites beaumonti* (d’Archiac & Haime). 2: *Nummulites gizehensis* (Forskal). 3: *Nummulites perforatus* (de Montfort). 4: *Nummulites discorbinus* (Schlotheim). (**c**) The exposed rock units at the El-Moniedra area, showing the Observatory Formation unconformably overlying the Galala Formation, the glauconite bed marked by the green lines.

The lower part of Minia Formation consists of white marl, grading into greyish-white, thinly-bedded marly limestone, and capped by cavernous limestone (Fig. 3a). The middle part is characterized by chalky limestone, intercalated with white sandy limestone, while the upper part is entirely made up of limestone (Fig. 2). Furthermore, Minia Formation yields several planktonic foraminiferal species, such as *Acarinina pentacamerata* (Subbotina) and *Turborotalia frontosa* (Subbotina). Therefore, Minia Formation can be assigned to the late Ypresian age. This is in alignment with the work of El-Dawoody and Galal^16^ that proposed a late Early Eocene (Ypresian) age for Minia Formation. Minia Formation conformably underlies the Middle Eocene Gebel Hof Formation (Fig. 2).

Gebel Hof Formation was proposed by Farag and Ismail^37^ to describe a 120-meter-thick section of white limestone, chalky at the base, and interspersed with thin bands of hard dolomitic limestone at Gebel Hof area, near Helwan City. In the study area, it is prominently exposed at the top of Wadi El-Qanaa area, where its thickness reaches about 130 m (Fig. 2).

The basal part of Gebel Hof Formation at Wadi El-Qanaa area consists of cherty limestone, followed by a thick marly limestone bed intercalated with limestone (Fig. 2). The uppermost part is made up of snow-white chalky limestone, creating a striking visual contrast (Fig. 2). Gebel Hof Formation at Wadi El-Qanaa area is assigned to the Middle Eocene (early-middle Lutetian) age according to El-Dawoody and Galal^16^, based on the presence of *Nummulites beaumonti* (de Montfort), *Nummulites discorbinus* (Schlotheim), and *Operculina* sp.

Observatory Formation was introduced by Farag and Ismail^37^ to describe an approximately 80-meter-thick section of white to golden-tan, marly, and nodular limestone at the Observatory Plateau, east of Helwan City. In the study area, it is recorded at El-Hamma area, with a thickness of about 15 m, and at El-Moniedra area, where its thickness reaches about 50 m (Fig. 2).

At El-Hamma area, Observatory Formation is characterized by highly fossiliferous limestone followed by a thin sandy limestone layer. The upper part consists of a distinctive oyster bed capped by white to yellow, cavernous limestone (Fig. 3b). Observatory Formation is characterized by rich faunal content, including significant elements such as oyster bivalves, gastropods, bryozoans, corals, echinoids, algae, and larger benthic foraminifera. Among the identified larger benthic foraminifera, *Nummulites gizehensis* (Forskal), *Nummulites discorbinus* (Schlotheim), *Nummulites beaumonti* (d’Archiac & Haime), and *Nummulites perforatus* (de Montfort) (Fig. 3b).

At El-Moniedra area, the lower part is made up of intercalations between limestone and dolomite (Fig. 3c). The middle part consists of a thick dolomite bed overlain by golden-tan, limestone, containing a green gluaconite bed. The upper part is characterized by a distinctive oyster bed capped by thin dolomite layer (Fig. 3c). Observatory Formation can be attributed to the Middle Eocene age based on the included larger benthic foraminifera, e.g., *Nummulite gizehensis* (Forskal), *Nummulite perforatus* (de Montfort), and *Nummulite beaumonti* (d’Archiac & Haime).

### Microfacies analysis

The microfacies analysis of the carbonate beds of Minia, Gebel Hof, and Observatory formations allowed the identification of seven microfacies types. The recognized microfacies types are compared with the standard microfacies types of Flügel^32^ and the facies zones of Wilson^33^.

**Fig. 4:**
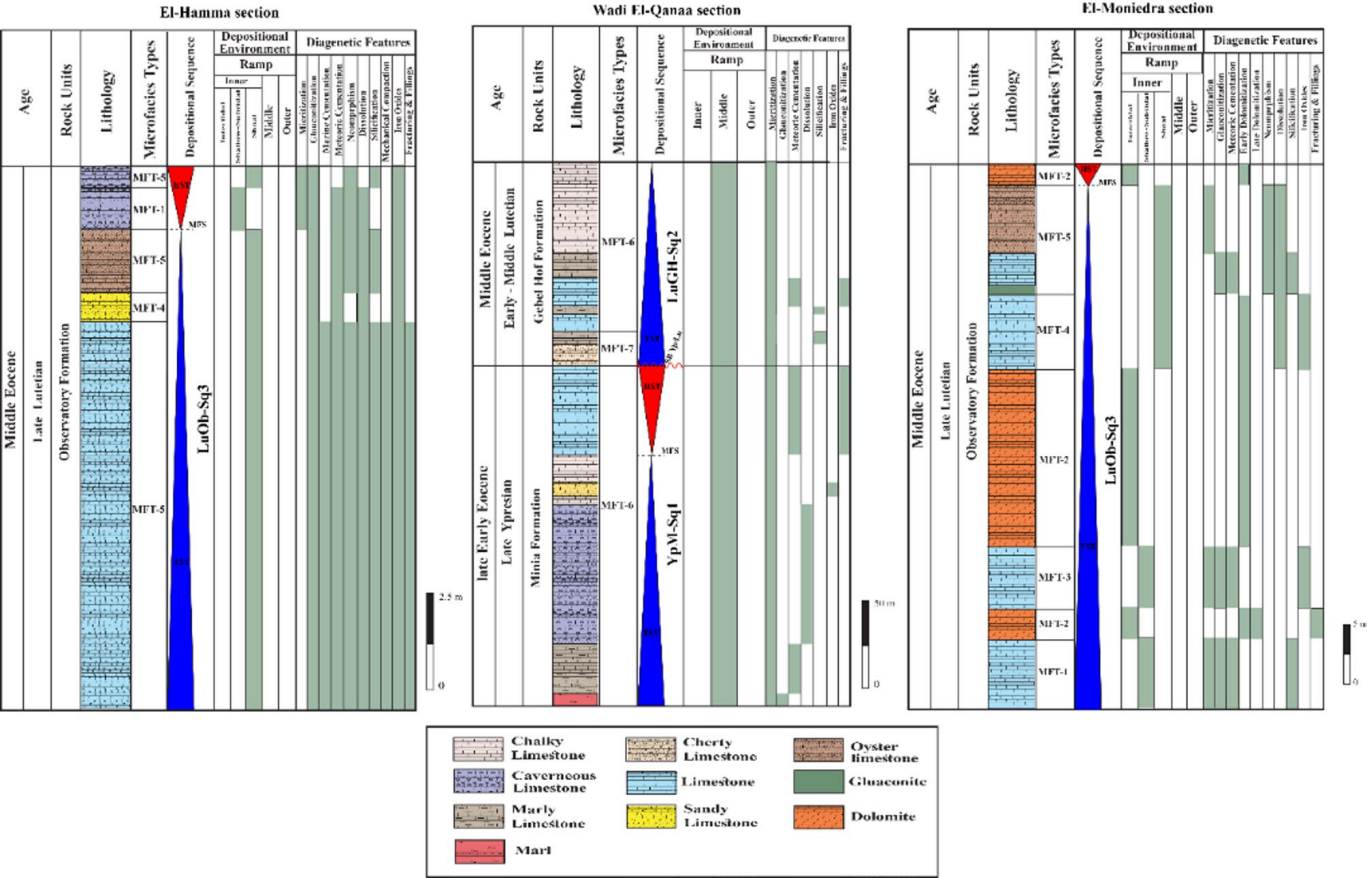
Lithostratigraphic sections from the study area showing the microfacies types, depositional sequences and environments, and the distribution of the common carbonate diagenetic features throughout the recognized rock units.

**Fig. 5:**
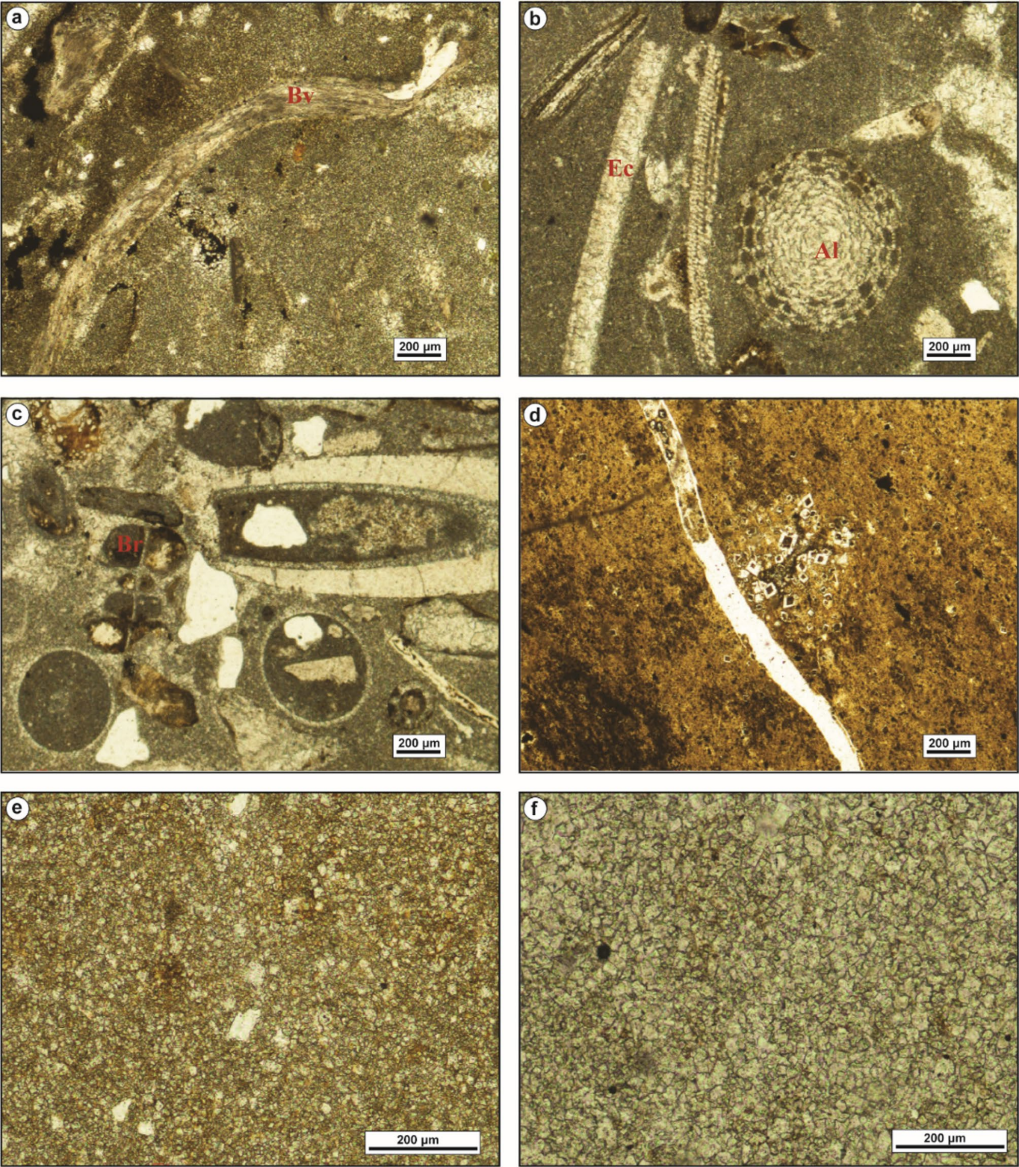
(**a**–**c**) Bioclastic floatstone (MFT-1), with bivalves (Bv), echinoids (Ec), algae (Al), and bryozoa (Br). (**d**–**f**) Ferruginous dolomite (MFT-2), showing medium-grained, zoned dolomite crystals, filling the dissolution pores.

The bioclastic floatstone microfacies (MFT-1) is recorded from the uppermost part of Observatory Formation at El-Hamma area and the lowermost part of Observatory Formation at El-Moniedra area (Fig. 4). It comprises rich faunal content with high diversity in microsparite cement, along with a few, fine to medium, subangular quartz grains, peloids, glauconite grains, and ferruginous patches (Fig. 5a–c). The faunal content includes bivalve oyster shells, gastropod shell fragments, serpulid worms, bryozoan fronds, miliolid and larger foraminiferal tests, coral fragments, echinoid plates, and various algal plates.

The ferruginous dolomite microfacies (MFT-2) is reported at various intervals within Observatory Formation at El-Moniedra area (Fig. 4). This microfacies consists of fine- to medium-grained, zoned, subhedral to euhedral dolomite crystals, with ferruginous material, iron oxides, and fine to medium, subangular quartz grains. Additionally, a few bivalve shell fragments are recorded (Fig. 5d–f).

**Fig. 6:**
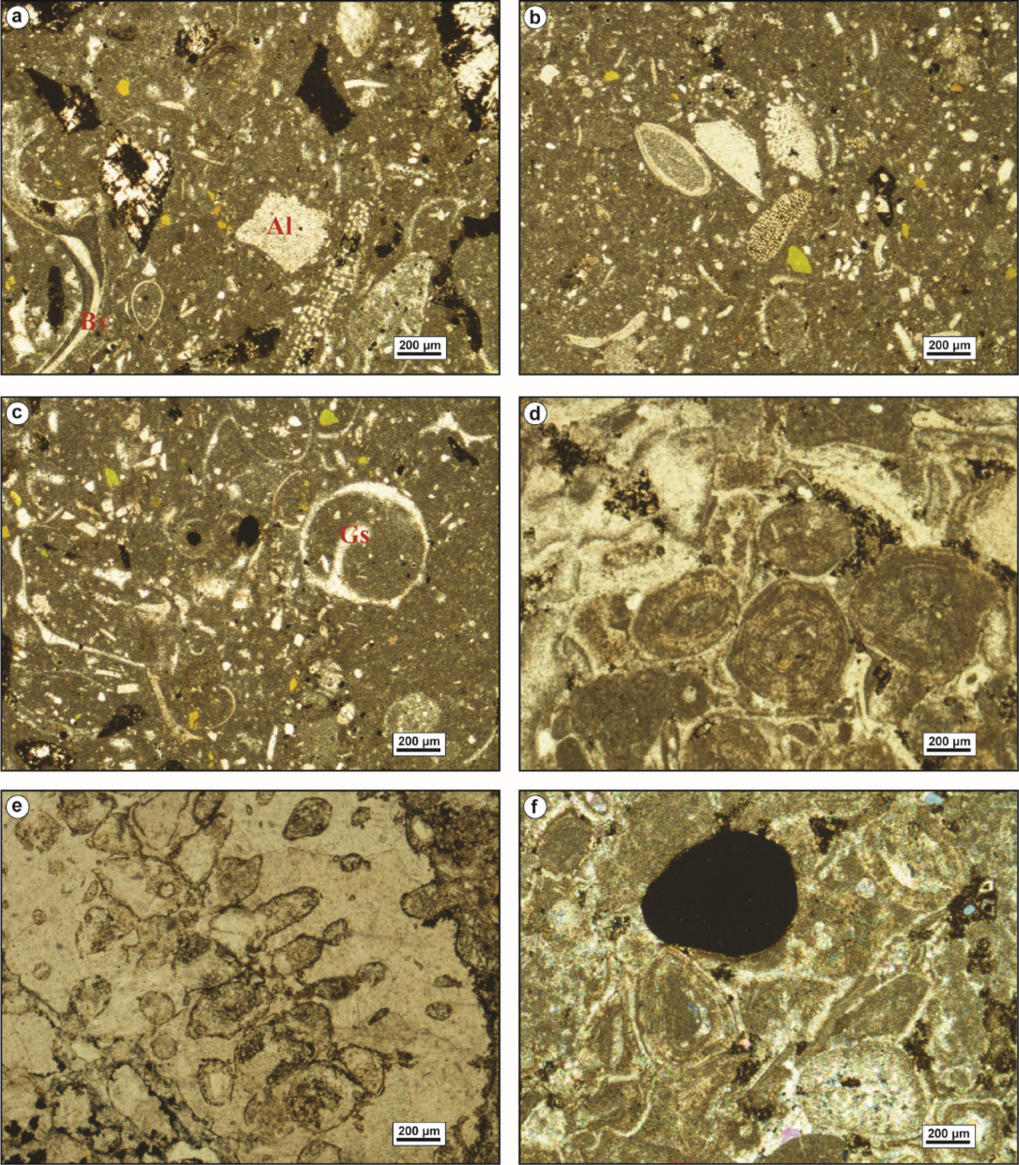
(**a**–**c**) Bioclastic packstone (MFT-3), with bivalves, gastropod shells (Gs), and algal plates. (**d**–**f**) Ferrigenous peloidal bioclastic grainstone (MFT-4**)**, with bryozoa (**e**) and phosphate grains (**f**).

The bioclastic packstone microfacies (MFT-3) is recorded from the lower part of Observatory Formation at El-Moniedra area (Fig. 4). The groundmass of this microfacies is represented by micrite cement, containing fine to medium, subangular quartz grains, yellowish glauconite pellets, ferruginous material, and some subhedral, zoned dolomite crystals (Fig. 6a–c).

Skeletal components include bivalve shell fragments, gastropod shells, and various algal plates, with some algae encrusting the bivalve shells.

The ferruginous peloidal bioclastic grainstone microfacies (MFT-4) is reported in the middle part of Observatory Formation at El-Hamma and El Moniedra areas (Fig. 4). This microfacies includes a highly diversified biota in a groundmass of microsparite. It has moderately-sorted, fine to medium, subangular to angular quartz grains, ooids, peloids, some subhedral, zoned dolomite crystals, phosphate grains, few ferruginous patches, glauconite grains, and iron oxides (Fig. 6d–f). The main skeletal components are bivalve shell fragments, gastropod shells, echinoid spines, bryozoan fronds, coral fragments, benthic foraminiferal tests, and various algal plates (Fig. 6e).

**Fig. 7:**
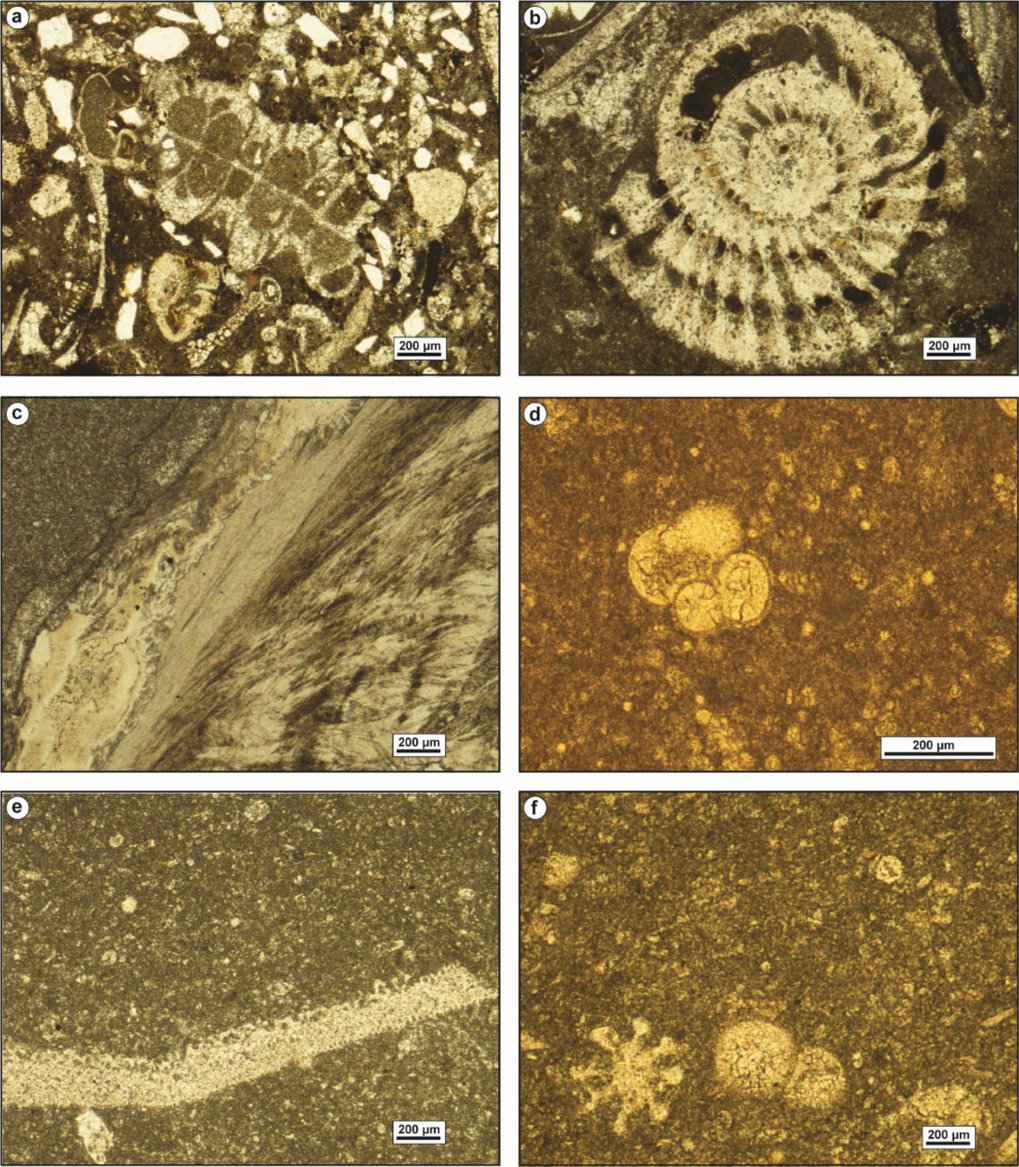
(**a**–**c**) Sandy peloidal bioclastic rudstone (MFT-5), with bryozoa, oyster bivalves, and the larger benthic foraminifer *Nummulite beaumonti* (d’Archiac & Haime) (b). (d-f) Foraminiferal wackestone (MFT-6), with *Acarinina pentacamerata* (Subbotina) (d) and echinoid fragments.

The sandy peloidal bioclastic rudstone microfacies (MFT-5) is recorded from various intervals within Observatory Formation at El-Hamma and El-Moniedra areas (Fig. 4). This microfacies is characterized by a highly diverse faunal content in microsparite cement, with a few ferruginous patches, ooids, peloids, fine subangular quartz grains, euhedral zoned dolomite crystals, glauconite grains, and a few phosphate grains (Fig. 7a–c). The faunal content consists of bivalve oyster shells, gastropod shells, coral fragments, echinoid plates with microbial encrustation, larger foraminiferal tests, bryozoan fronds, and various algal plates.

The foraminiferal wackestone microfacies (MFT-6) is recorded from the lowermost part of Gebel Hof Formation at Wadi El-Qanaa area (Fig. 4). This microfacies is made up of a highly diversified biota in a groundmass of micrite. The main skeletal components are planktonic and benthic foraminiferal tests, including miliolids, bivalve and gastropod shells, echinoid plates and spines, serpulid worms, and various algal plates (Fig. 7d–f).

**Fig. 8:**
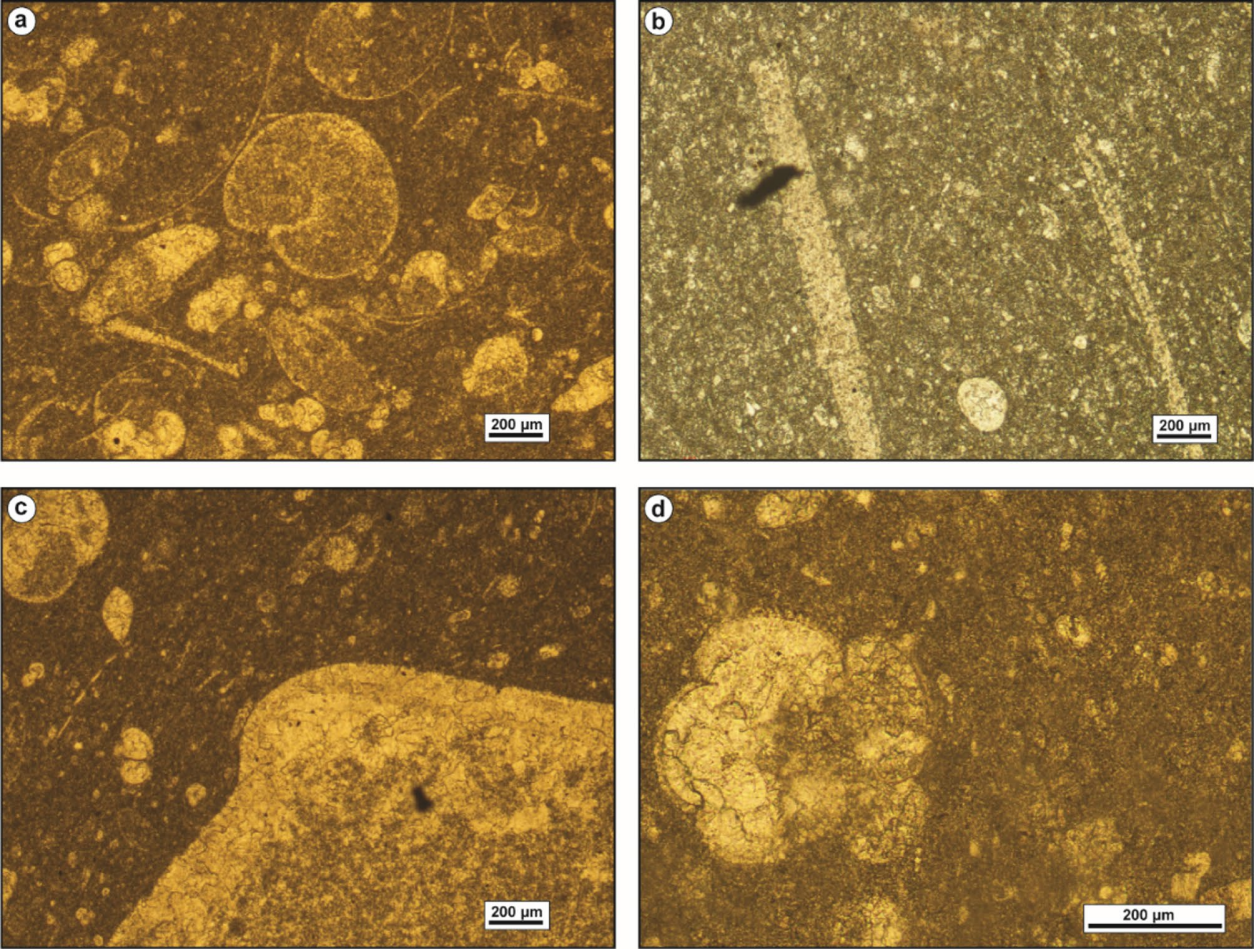
Bioclastic packstone (MFT-7), with bivalves, echinoids, and planktonic foraminifera.

The bioclastic packstone microfacies (MFT-7) is recorded from various intervals within Minia Formation and from the middle and upper parts of Gebel Hof Formation at Wadi El-Qanaa area (Fig. 4). The groundmass of this microfacies consists of micrite cement, with skeletal components including planktonic and benthic foraminiferal tests, gastropod shells, and bivalve shell fragments (Fig. 8).

### Sequence stratigraphy

The sequence stratigraphy of the study area, which spans from the late Early to Middle Eocene, was developed by integrating lithostratigraphic observations with detailed microfacies analysis, revealing important insights into the relative sea-level changes and accommodation space variations during the deposition of the Minia, Gebel Hof, and Observatory formations.

Minia Formation: This formation corresponds to the Transgressive Systems Tract (TST) of the first depositional sequence (YpM-Sq1; Fig. 4). The lower part, composed of white marl and marly limestone, reflects low-energy outer ramp conditions, while the middle section, marked by chalky limestone, indicates increased carbonate production during sea-level rise, reaching the maximum flooding surface (MFS). The upper part transitions to the early Highstand Systems Tract (HST), reflecting shallowing-upward conditions as accommodation space decreased.

A well-defined sequence boundary (SB Yp/Lu) separates the Minia Formation (YpM-sq1) and the overlying Gebel Hof Formation (LuGH-sq2) in Wadi El Qanaa area. This boundary is marked by a sharp lithological shift from the HST of YpM-sq1 (shallowing-upward pure limestone) to the basal cherty limestone of LuGH-sq2 (Fig. 4). The SB reflects a fall in relative sea level, culminating in the exposure of the carbonate platform and a basinward shift in facies prior to the renewed transgression recorded in LuGH-sq2. This boundary aligns with regional Late Ypresian–Early Lutetian eustatic fluctuations, emphasizing the interplay between tectonics and eustasy in controlling accommodation^1^.

Gebel Hof Formation: This formation represents the Highstand Systems Tract (HST) of the second depositional sequence (LuGH-Sq2; Fig. 4). The basal cherty limestone reflects mid-ramp deposition with episodic siliciclastic influx, transitioning upward into marly limestone, indicating a shallowing trend. The upper part, dominated by pure limestone, represents a low-energy lagoonal to shoal environment, marked by benthic foraminiferal assemblages.

Observatory Formation: The Observatory Formation includes both the Transgressive Systems Tract (TST) and Highstand Systems Tract (HST) within the third sequence (LuOb-Sq3; Fig. 4). At the El-Hamma area, highly fossiliferous limestone marks the initial transgression, while sandy limestone reflects increased siliciclastic input. The upper oyster beds and cavernous limestone signify a reduced accommodation space and regression, consistent with the HST. At the El-Moniedra area, glauconite-rich layers and ferruginous dolomites indicate a maximum flooding event followed by shoaling and progradation.

### Carbonate diagenetic analysis

Petrographical analysis of the studied carbonate rocks revealed multiple diagenetic processes, altering their original depositional textures, such as micritization, glauconitization, dolomitization, and neomorphism (Fig. 4; Table 1). Micritization refers to the process whereby carbonate allochems undergo replacement by crypto- and microcrystalline calcite, leading to a gradual obliteration of their original textures^38,39^. This phenomenon is most pronounced in the shallow subtidal, including shoal, inner ramp carbonate sediments of Observatory Formation at El-Hamma and El Moniedra areas (Fig. 4).

#### Micritization

**Fig. 9:**
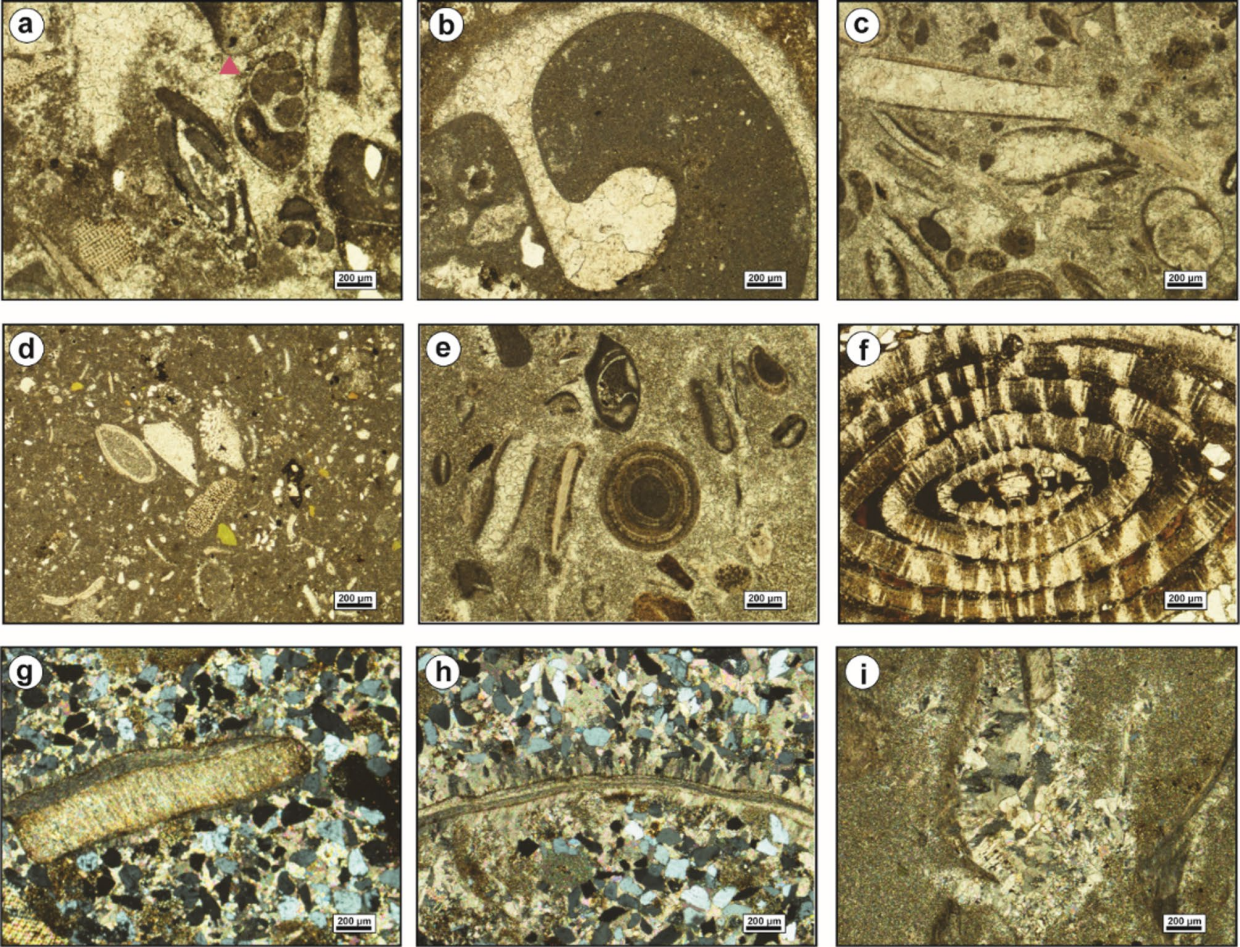
(**a**) and (**b**) Thin-section photomicrographs show the micritization of a gastropod shell. (**c**) Micrite envelope around the periphery of bivalves and echinoidal plates. (**d**) Glauconitic grains scattered in a micrite matrix. (**e**) Glauconites partially replace ooids. (**f**) Infilling of a nummulite chamber by glauconites. (**g**) and (**h**) Thin-section photomicrographs demonstrate fibrous calcite cement. (**i**) Filling of a dissolution pore by granular spar cement.

Skeletal particles such as echinoderms and mollusks exhibit various degrees of susceptibility to micritization. This process entails the partial replacement of their internal structures by microstructure-controlled minute patches of dark-colored micrite. Despite these alterations, the overall size, outer margins, and remnants of the internal structures of micritized fossils remain discernible (Fig. 9a, b). Micritization also led to the formation of micrite envelopes, typically 18–46 μm-thick, outlining the peripheries of bivalve shells and echinoid plates (Fig. 9c). In these instances, micritization initiated from the outer margins of the skeletal grains and progressed inward toward the center. These micrite envelopes serve to stabilize the carbonate grains, preventing further diagenetic alterations.

#### Glauconitization

Glauconites are volumetrically-infrequent in the analyzed carbonates. The glauconitic grains are dispersed throughout the matrix (Fig. 9d). They are prevalent in the shallow subtidal inner ramp carbonates of Observatory Formation at El-Hamma and El Moniedra areas (Fig. 4). The glauconites exhibit yellowish to reddish-green color, poor to moderate sorting, and subrounded to subangular shape. Additionally, glauconite partially replaces ooids and larger foraminifera (Fig. 9e, f).

#### Calcite cementation

Calcite cement is the most prevalent cement type in the examined carbonates, exhibiting numerous fabric varieties that reflect diverse diagenetic environments. Fibrous calcite cement is uncommon in the studied carbonates due to its deterioration by compaction processes over time. It has been identified in the shallow subtidal inner ramp carbonates of Observatory Formation at El-Hamma area (Fig. 4). The fibrous calcite cement is composed of fine-crystalline, needle-shaped, planar, tightly-packed calcite crystals, with translucent to cloudy fringes (Fig. 9g, h).

Sparry calcite cement represents the most significant porosity-destructive cement. It displays two main fabrics; granular spar and blocky calcite. The granular spar occupies interparticle pore spaces, consisting of inclusion-free, transparent, subhedral to anhedral crystals (Fig. 9i). Blocky calcite cement refers to limpid, transparent, mud-free, subhedral, non-equidimensional, and coarse calcite crystals with distinct planar intercrystalline boundaries.

**Fig. 10:**
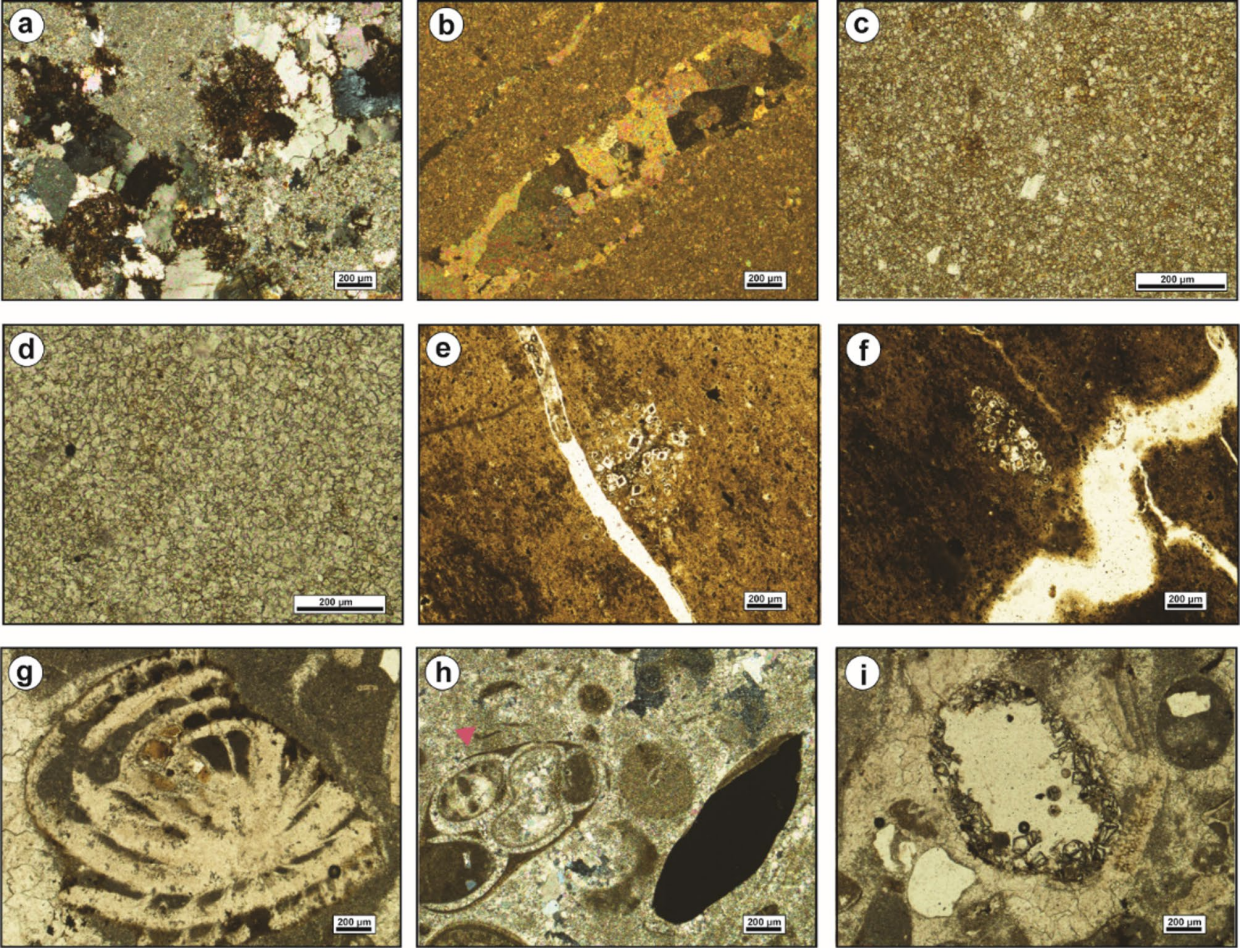
(**a**) Blocky calcite cement fills a pore-space formed by dissolution of matrix. (**b**) Thin-section photomicrograph displays granular calcite fills a microfracture. (**c**) and (**d**) Thin-section photomicrographs show the fine-crystalline, replacive dolomites. (**e**) and (**f**) Thin-section photomicrographs display the occlusion of open void by medium-grained, zoned, euhedral dolomite cement. (**g**) Aggrading neomorphism of the micitic matrix into neomorphic spar. (**h**) Thin-section photomicrograph shows the neomorphic calcitization of gastropod shell into inclusion-rich, granular neomorphic spar. (**i**) The partial infilling of a void space by quartz cement.

Two phases of blocky calcite cement were identified in the studied carbonates. The earlier phase fills solution pore spaces, with crystal size increasing towards the center of the pore space (Fig. 10a). This phase is commonly observed in the shallow subtidal inner ramp carbonates of Observatory Formation at El-Moniedra and El-Hamma areas, as well as in the middle ramp carbonates of Minia and Gebel Hof formations at Wadi El-Qanaa area (Fig. 4). The second phase consists of blocky crystals with undulatory extinction occurring within fractures (Fig. 10b). It is mainly recorded in the shallow subtidal inner ramp carbonates of Observatory Formation at El-Moniedra area (Fig. 4).

#### Dolomitization

The petrographical investigation of the recorded dolomites reveals two modes of emplacement; replacive dolomites and void-filling dolomites. The matrix replacive dolomites are gray to yellowish-gray, hard, and burrowed. They are fine- to very fine-crystalline, displaying primary intercrystalline porosity (Fig. 10c, d).

These dolomites show uniform extinction, and unimodal crystal size distribution. Most of these dolomites are unzoned, exhibiting cloudy cores due to inclusions of precursor lime mud relicts. This phase is commonly observed in the intertidal and shallow subtidal shoal inner ramp carbonates of Observatory Formation at El-Hamma and El-Moiendra areas (Fig. 4).

Void-filling dolomite shows limited distribution in the studied carbonates. It is identified as a filling material that partially to entirely occludes open voids and dissolution pores. Dolomite cement has been observed in the intertidal carbonates of Observatory Formation at El-Moniedra area (Fig. 4). The examined dolomite cement is characterized by fine to medium crystalline, well-developed, idiomorphic dolomite rhombs and undulose extinction. Most of these rhombs are distinctly zoned, featuring dark reddish iron-oxide rhombic cores with clear rims, while some are completely limpid (Fig. 10e, f).

#### Neomorphism

Aggrading neomorphism of the micritic matrix is a prominent characteristic in the studied mud-supported carbonates (Fig. 4). This process involves the recrystallization of fine-crystalline micritic matrix into coarser neomorphic spar (Fig. 10g). The neomorphic spar consists of impure, granular, patchy, anhedral calcite crystals with curved to irregular intercrystalline contacts. It typically retains small remnants of the original unreplaced micritic matrix.

Neomorphic calcitization of skeletal allochems is a stabilization process that affects the bioclasts of gastropods and bivalves in different degrees, resulting in partial to complete obliteration of their original structures. During the progression of neomorphic calcitization, the internal structures of skeletal allochems are replaced by brownish-colored, non-planar, roughly-distributed granular calcite mosaics with curved boundaries (Fig. 10h).

Aggrading neomorphism of matrix and neomorphic calcitization of skeletal allochems are both documented in the shallow subtidal, including shoal, inner ramp carbonates of Observatory Formation at El-Hamma and El-Moiendra areas (Fig. 4).

#### Silicification

**Fig. 11:**
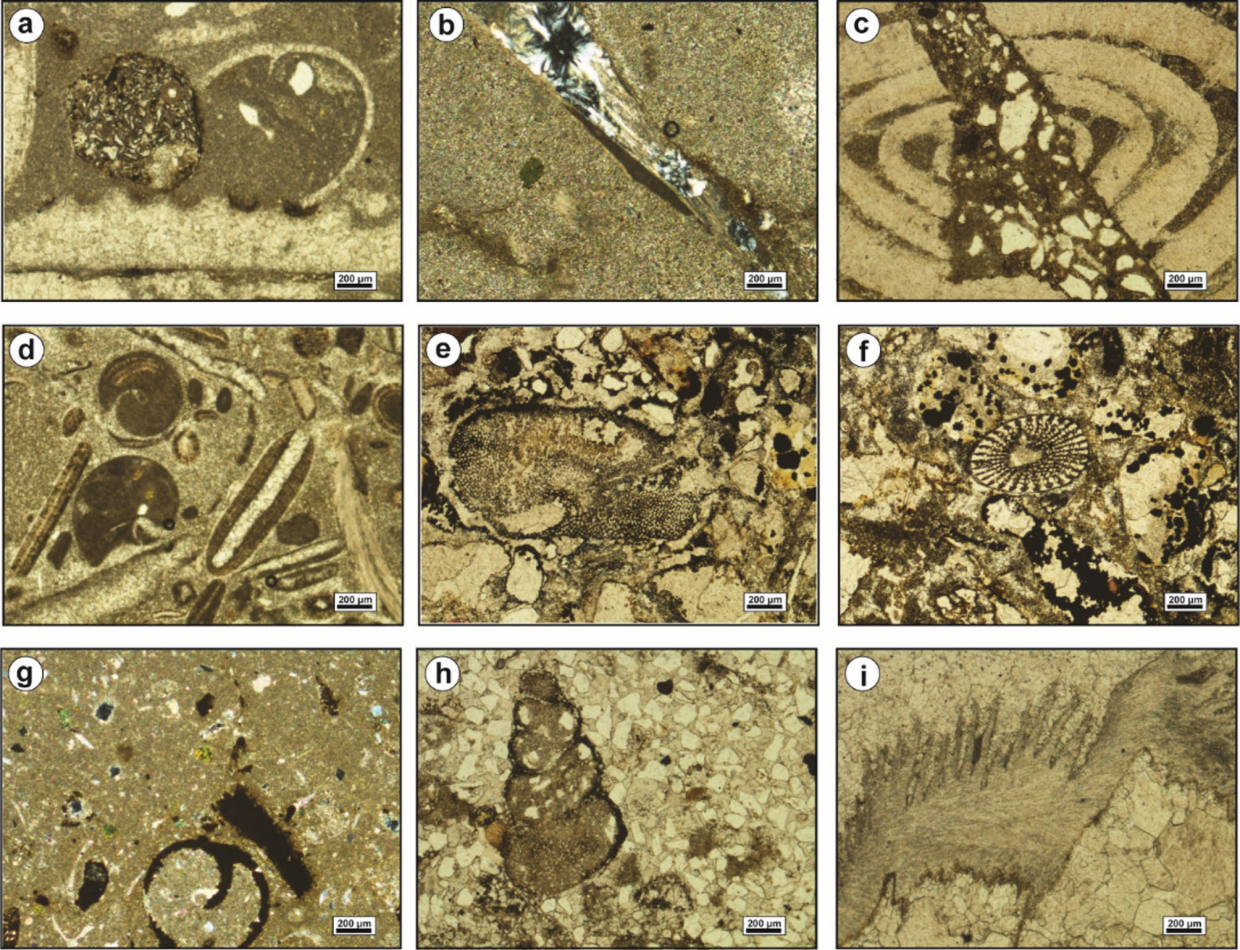
(**a**) Quartz cement fills a cavity in micrite matrix. (**b**) Quartz cement fills biomoldic porosity of bivalve shell formed by dissolution of its internal structure. (**c**) A fracture in a nummulitic grain due to mechanical compaction. (**d**) Thinsection photomicrograph displays rearrangement of grains as result of mechanical compaction. (**e**) and (**f**) Iron-oxide replaces the matrix. (**g**) and (**h**) Iron-oxide envelope around the periphery of gastropod shells. (**i**) Moldic pore formed as result of selective dissolution of the calcite cement.

Silica is present as a cement filling pore spaces in the middle ramp carbonates of Minia and Gebel Hof formations at Wadi El-Qanaa area and the shallow subtidal shoal inner ramp carbonates of Observatory Formation at El-Hamma and El-Moiendra areas (Fig. 4). The silica cement in these rocks consists of fine to medium, anhedral quartz crystals, and dispersed throughout the matrix (Fig. 9g, h). The quartz crystals partially to entirely occlude open voids and dissolution pores (Figs. 10i and 11a). Some skeletal particles such as bivalves show partial replacement of their internal structures by silica (Fig. 11b).

#### Mechanical compaction

Compaction is a post-depositional process resulting from a progressive increase in burial load or from crustal tectonic stresses^32^. It leads to the loss of interparticle porosity, dewatering, and reduction in bed thickness. The style of compaction is primarily influenced by rock texture, mineral stabilization, sedimentation rate, cementation, and overburden burial depth^14^. The mechanical compaction involves grain rearrangement, contortion, fracturing, and breakage of allochemical constituents (Fig. 11c, d). This phenomenon is most pronounced in the shallow subtidal shoal inner ramp carbonates of the Observatory Formation at El-Hamma and El-Moiendra areas (Fig. 4).

#### Iron oxides

The presence of iron oxides is quite limited in the examined rocks. It is recorded in the shallow subtidal shoal inner ramp carbonates of Observatory Formation at El-Hamma and El-Moiendra areas (Fig. 4). It manifests as brownish to reddish-black pockets and patches that impregnate the matrix, replace shells, and coat some allochemical constituents (Fig. 11e–h). Algae and echinoid plates are particularly susceptible to iron-oxide pigmentation.

#### Fracturing

Fractures are sparsely distributed throughout the studied sequence, observed at both macroscopic and microscopic scales (Figs. 10b, e and f and 11c). Macroscopic fractures were identified within the intertidal and shallow subtidal shoal inner ramp carbonates of Observatory Formation at El-Hamma and El-Moniedra area, and in the middle ramp carbonates characterizing Minia and Gebel Hof formations at Wadi El-Qanaa area (Fig. 4).

#### Dissolution

Dissolution is a prevalent feature observed in the studied carbonates. It impacts the matrix, cement, and skeletal allochems, leading to the obliteration of original textures and the formation of secondary porosity^40^. This process occurs as carbonate constituents are leached out by diagenetic fluids that are undersaturated with respect to CaCO_3_^41,42^. This phenomenon is most pronounced in shallow subtidal inner ramp carbonates of Observatory Formation at El-Moniedra and El-Hamma areas (Fig. 4). As a result of the selective dissolution of cement and skeletal allochems, leaving behind open moldic pores, many pores are later filled or partially occluded by meteoric or burial cements during subsequent diagenetic stages (Figs. 9c and e and 11i).

## Discussion

### Depositional model: paleoenvironmental interpretation of the studied carbonates

The depositional model developed for the studied carbonate ramp system reflects a transition from high-energy inner ramp settings to lower-energy middle ramp environments, providing insights into the depositional dynamics and diagenetic imprints of the area. The vertical and lateral transitions observed in the studied facies, coupled with the absence of abrupt breaks or significant reefal barriers, which are hallmarks of ramp systems. The ramp in this study is interpreted as a homoclinal ramp, typified by a gently sloping profile and dominated by high-energy shoals in the inner ramp transitioning into lower-energy middle ramp settings. These ramp settings were previously discussed by several authors^5,9,25,43–46^.

Tectonic activity and sea-level changes played significant roles in shaping this depositional system. The Syrian Arc tectonism during the Eocene caused subtle subsidence and uplift, influencing accommodation space and sedimentation rates^47^. Eustatic sea-level fluctuations further modulated the depositional environments, promoting transgressive-regressive cycles that are evident in the facies stacking patterns and transitions between inner and middle ramp settings^5^.

#### Inner ramp depositional settings

**Fig. 12:**
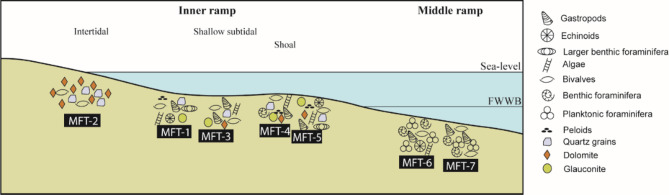
Paleoenvironmental model for the Lower–Middle Eocene successions in the study area, with the distribution of the recognized microfacies types.

Ferruginous peloidal bioclastic grainstone microfacies (MFT-4) and sandy peloidal bioclastic rudstone microfacies (MFT-5): These facies exhibit characteristics of high-energy environments, including the presence of ooids, peloids, and abundant faunal diversity (e.g., foraminifera, bivalves, echinoids). Microsparitic cement and well-sorted grains further affirm deposition in a shallow subtidal shoal setting (Fig. 12). The high-energy dynamics align with SMF-11 and SMF-12-S of Flügel^32^ and Facies Zone (FZ-6), Platform Margin Shoal of Wilson^33^. These settings likely developed during periods of sea-level highstand when high-energy conditions prevailed on the inner ramp.

Bioclastic floatstone microfacies (MFT-1) and bioclastic packstone microfacies (MFT-3): These facies indicate deposition in a shallow, open-marine environment, as evidenced by glauconite pellets, peloids, and diverse faunal assemblages. Microsparitic cement and high-energy indicators suggest a dynamic shallow subtidal setting (Fig. 12), correlating with RMF-15 and SMF-12 of Flügel^32^ and Facies Zone (FZ-8), Open-Marine Platform Interior of Wilson^33^. Tectonic stability and steady sea levels likely led to these open-marine conditions.

Ferruginous dolomite microfacies (MFT-2): This facies reflects deposition under restricted circulation conditions, as indicated by fine dolomite crystals, ferruginous material, and the absence of faunal components. These characteristics are typical of peritidal environments (Fig. 12), correlating with SMF-23 of Flügel^32^ and Facies Zone (FZ-8), Restricted Platform Interior of Wilson^33^. Restricted settings may result from local tectonic uplift or periods of relative sea-level fall, reducing water depth and circulation.

#### Middle ramp depositional settings

Foraminiferal wackestone microfacies (MFT-6) and bioclastic packstone microfacies (MFT-7): These microfacies are indicative of deeper, low-energy middle ramp settings. The dominance of micritic cement, planktonic foraminifera, and skeletal grains points to open circulation in a calm marine environment (Fig. 12). These facies correspond to RMF-9 and RMF-7 of Flügel^32^. Subsidence associated with tectonic activity likely created the accommodation space necessary for these low-energy environments, while periods of sea-level rise facilitated their development.

###  Regional correlation of depositional sequences

The sequence stratigraphic framework of the study area aligns with observations from similar Eocene carbonate successions across the Eastern Mediterranean. The Minia Formation’s TST corresponds with similar transgressive deposits in the Northern Galala Plateau^14^ and the Eastern Desert, reflecting global eustatic sea-level rise during the Late Ypresian^46^. The Gebel Hof Formation’s HST mirrors Lutetian carbonate platform dynamics described in various Egyptian basins^1^. The Observatory Formation’s glauconitic layers align with Middle Eocene maximum flooding surfaces identified in the Nile Valley and North African basins^48–50^, underscoring the significance of transgressions during the Lutetian.

This integrated depositional and sequence stratigraphic model highlights the interplay of tectonics, eustatic sea-level changes, and sedimentary processes in shaping the carbonate successions of the North Eastern Desert. It provides a comprehensive understanding of the paleoenvironmental evolution and diagenetic history of carbonate systems, applicable to similar geological settings in the region.

###  Diagenetic interpretation

Micritization typically occurs within a marine-phreatic diagenetic environment shortly after deposition, either at or just beneath the sediment-water interface under tranquil water conditions^42,51–53^. Consequently, the micritization of allochems in Observatory formation at El-Hamma area is indicative of a shallow subtidal shoal environment characterized by relatively slow sedimentation rates. The presence of micrite-filled microborings on the peripheries of the micritized grains suggests a bioerosion origin for the micrite envelopes (Fig. 9c).

The primary prerequisites for glauconitization include suitable substrates, supply of Fe and K, sub-oxic and mild reducing conditions, a confined micro-environment, and a slow sedimentation rate^54^. Potential substrates for glauconitization comprise biogenic materials (e.g. fossil bioclasts and fecal pellets), detrital minerals (e.g. quartz, biotite, feldspars, mica, and phosphatic collophane), and clay minerals^14^. Restricted conditions facilitate glauconitization by stabilizing lime-mud, which acts as a substrate for the glauconitization of fecal pellets^54^. Additionally, the lack of mechanical abrasion supports the authigenic origin of the identified glauconitic grains^14^.

Textural evidence indicates that the studied glauconites likely formed through the replacement of lime-mud within nummulite chambers or as part of the matrix^55^. The entrapment of glauconites within skeletal particles (nummulites) and the presence of irregular glauconitic patches in the matrix reinforce the diagenetic authigenic origin of these glauconites [Fig. 9f and^14^].

The presence of fibrous calcite cement in shallow subtidal environment, its localized precipitation on the peripheries of fossil bioclasts, and its sharp contact with the surrounding allochems and orthochems suggest its formation during early diagenesis in the marine-phreatic diagenetic realm^56,57^. Syntaxial overgrowth cement has been observed to precipitate in both marine and meteoric diagenetic environments^38,56,58–60^.

The identified syntaxial overgrowth cement is likely an early marine-phreatic diagenetic precipitate, as indicated by its cloudy, inclusion-rich appearance and its presence in mid-ramp environment, where marine cementation predominates^14,61^.

The sparry calcite cement, composed of Low-Mg calcite, precipitates in both meteoric environments and burial environments^62–64^. Additionally, Melim et al.^65^ and Ahmad et al.^66^ documented sparry calcite formation in marine-phreatic fluids. The textural relationships, inclusion-free nature, equant-drusy fabric, and lack of compactional features suggest that the first phase of sparry calcite cement precipitated in a meteoric-phreatic environment following the dissolution of skeletal allochems and lime mud matrix^14,38,67^ The occurrence of the second phase of sparry calcite cement within fractures indicates its formation during late-stage diagenesis (i.e., deep burial or meteoric-vadose diagenesis following emergence due to uplift).

The syngenetic origin of the studied replacive dolomites is evidenced by several characteristics; (1) the dolomitization of the lime mud matrix, indicating formation during early diagenesis before the mineralogical stabilization of the matrix into low Mg-calcite^67^, (2) the fine crystal size and sporadic distribution of these dolomites^68^, (3) the euhedral form and unimodal size distribution^67^, and (4) the unzoned habit and inclusion-rich cores^69^.

Normal to slightly saline seawater is considered the most suitable solution for dolomitization during the early stages of diagenesis^66^. Restricted intertidal limestones can undergo dolomitization influenced by normal to slightly saline water, resulting in dolomites without necessitating evaporite precipitation^66^. The absence of evaporites, along with the fine crystal size and textural characteristics of the studied replacive dolomites, indicate a non-hypersaline, shallow marine environment^70^.

Consequently, the Observatory matrix-replacive dolomites likely formed through seawater dolomitization of the precursor lime mud matrix in a marine-phreatic diagenetic environment during early diagenesis^63,71^. Tidal pumping of normal to slightly saline seawater^72,73^ or reflux of mesosaline marine fluids^74^ are the most likely mechanisms driving seawater through the studied lime mudstones, resulting in the formation of replacive dolomites.

The mechanism of neomorphism involves the in-situ conversion of metastable aragonite and high Mg-calcite, which constitute the matrix and skeletal allochems, into the more stable low Mg-calcite^55,75,76^. The presence of Mg ions inhibits the growth of CaCO_3_ crystals^77^. Therefore, the removal of Mg ions from high Mg-calcite or aragonite is crucial for the enlargement of these crystals. Numerous studies have recognized the role of meteoric freshwater and clay minerals in promoting aggrading neomorphism^14^.

Freshwater can dilute Mg ions, facilitating the conversion of aragonite and high Mg-calcite to low Mg-calcite and advancing the neomorphic process. During diagenesis, the expelled Mg ions are adsorbed onto the surfaces of clay minerals and/or argillaceous material, reducing the Mg content and promoting the growth of fine CaCO_3_ crystals. This evidence supports the meteoric-phreatic realm as the most likely diagenetic environment for the neomorphic recrystallization of the examined carbonates.

The general mechanism of silicification relies on the dissolution of carbonates and the subsequent precipitation of silica^78^. This process often occurs in environments with low pH and high CO_2_ concentrations^64^. Shaaban^79^ highlighted the role of meteoric water in lowering pH values and increasing CO_2_ concentrations. Silica cement is most likely precipitated through the influence of meteoric-phreatic water during regression periods^14,80^.

Tawfik et al.^13^ proposed that void-filling quartz precipitates in an open system with respect to CO_2_, where the solutions are more diluted in silica and not undersaturated with carbonates, leading to direct quartz precipitation as a filling material. The absence of siliceous bioclasts excludes a biogenic source for the silica. Additionally, the lack of pyroclastics and volcanogenic rock fragments rules out submarine volcanism as a source. Therefore, the present silica is most likely derived from terrestrial sources during periods of sea-level fall^13^.

Mechanical compaction initiates shortly after deposition and continues throughout burial to depths of several hundred meters^44^. At these depths, the pressure and temperature conditions are insufficient to precipitate carbonate cement^79^. Initial stages of mechanical compaction occur shortly after deposition in marine-phreatic diagenetic environments^79^, while later stages occur during shallow burial diagenesis^81,82^.

Presence of iron oxides primarily occurs above the water table in warm, oxidized environments associated with arid climates^51^. In such conditions, Fe ions gradually convert into iron oxide, likely hematite, over time. Therefore, the iron oxide observed in the studied carbonates likely represents an epidiagenetic process related to subaerial meteoric-vadose diagenesis during uplifting and emergence periods^13^. The iron necessary for this process may originate from various sources, including Fe-rich ascending hydrothermal solutions, iron-bearing minerals, subaerial weathering, and diagenetic alteration of clay minerals^13,14^. Additionally, Moore^40^ proposed that iron released during the aggrading neomorphism of the micritic matrix could serve as another source for iron.

The absence of volcanic activity in the study area rules out Fe-rich ascending hydrothermal solutions as a significant source for iron. Other potential sources of iron are indicated by; (1) the presence of iron-bearing minerals such as glauconite, (2) the occurrence of detrital quartz in the shallow subtidal carbonates of Observatory formation, reflecting the influence of subaerial exposure and weathering as a source of iron, and (3) the dominance of aggrading recrystallization processes in the studied carbonates.

### Relationship between diagenetic episodes and sequence stratigraphy

The depositional environments significantly influenced subsequent diagenetic modifications. High-energy inner ramp settings with active water circulation facilitated early cementation and micritization, while middle ramp environments, characterized by slower sedimentation rates, promoted dissolution and neomorphism. The restricted conditions at the platform margin provided favorable environments for dolomitization and glauconitization. These processes, tied to the depositional settings, collectively shaped the diagenetic history of the studied successions. By incorporating a detailed depositional model, this study provides a comprehensive understanding of the interplay between depositional processes and diagenetic imprints, offering insights into the paleoenvironmental evolution of carbonate systems in similar geological settings.

**Fig. 13:**
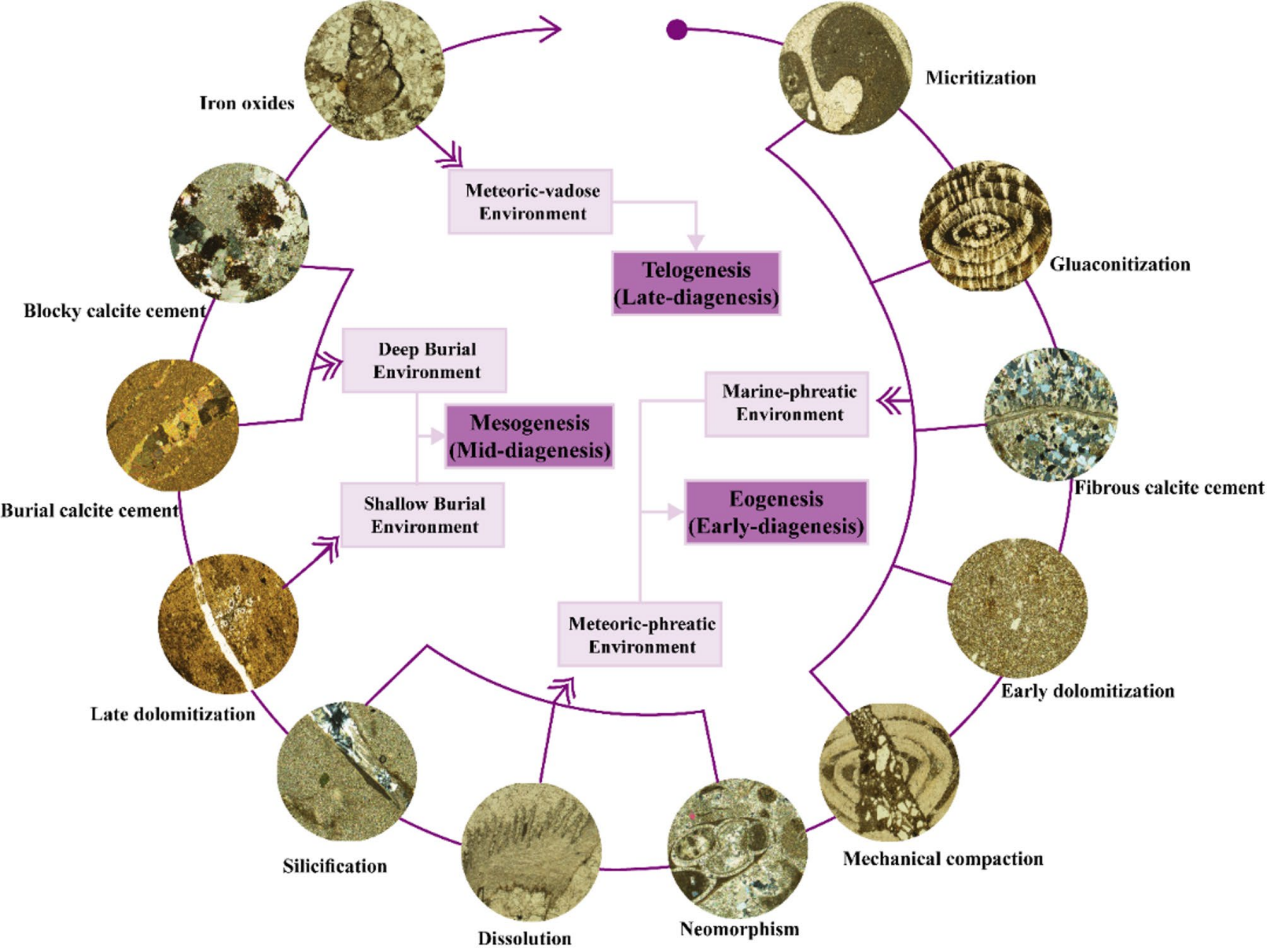
Generalized paragenetic scheme showing the reported diagenetic features and processes, and the interpreted diagenetic environments.

The examined carbonate rocks exhibit a complex diagenetic history, characterized by early, middle, and late diagenetic processes. Petrographical and microfacies analyses reveal that these diagenetic processes are associated with four distinct environments: marine-phreatic, meteoric-phreatic, burial, and uplift. These environments can be grouped into three sequential diagenetic episodes: eogenesis, mesogenesis, and telogenesis (Fig. 13). In the context of sequence stratigraphy, these diagenetic processes are influenced by changes in accommodation space and relative sea level fluctuations, which regulate the spatial and temporal distribution of diagenetic alterations.

Eogenetic processes encompass near-surface early diagenetic changes affecting carbonate sediments immediately post-deposition and pre-burial^83^. This realm includes both marine and meteoric diagenetic environments^14,84^. Marine-phreatic diagenesis involves several sequential processes: micritization, glauconitization, marine cementation, early dolomitization (matrix-replacive dolomites), and the initial stages of mechanical compaction. Marine-phreatic diagenesis occurs predominantly during transgressive and highstand systems tracts, where accommodation space is increasing and high-energy depositional settings promote early cementation and micritization processes. The microfacies analysis reveals that micritization represents the earliest diagenetic alteration in the studied carbonates, occurring contemporaneously with or slightly before marine cementation and subsequent dolomitization^38,63^. Glauconitization follows micritization as indicated by the replacement of ooids and micritized nummulites by glauconites [Fig. 9e and f and^85^]. This supports the interpretation of early marine-phreatic diagenesis, where microfacies change from more intact, coarse-grained structures to finer, altered materials.

The precipitation of fibrous marine cement on fossil allochem rims marks the first cement generation in the studied carbonates, occurring post-micritization and pre-compaction [Fig. 9h and^79^]. Petrographical analysis reveals that replacive dolomites formed during early diagenesis by substituting precursor lime mud in the marine-phreatic zone. Microtextural evidence from the microfacies further shows that replacive dolomites postdate marine-phreatic calcite cement^69^. Intercrystalline porosity between replacive dolomite rhombs indicates that dolomitization occurred prior to significant compaction [Fig. 10c and d and^86^]. Syndepositional compaction, evidenced by broken and deformed micritized grains (nummulites), developed during the late stages of marine-phreatic diagenesis (Fig. 11c)^65,85,86^.

This study identifies two phases of meteoric diagenesis, with microfacies analysis providing essential details of the shifts in pore space and mineralogy during these stages. The first phase, occurring after deposition and prior to burial, falls within early diagenesis (Eogenesis) and involved carbonate rocks’ exposure to meteoric fluids following sea-level drops (meteoric-phreatic diagenesis). The first meteoric phase is closely linked to the lowstand systems tract (LST), where exposure during relative sea-level fall triggers meteoric diagenesis, such as dissolution and neomorphism. The second phase occurred post-uplift during late diagenesis (Telogenesis), when uplifted strata were exposed to meteoric-vadose conditions. Meteoric-phreatic diagenesis includes neomorphism, dissolution, carbonate meteoric cementation, and silicification. Prolonged exposure to meteoric water results in metastable micritic matrix and skeletal particle leaching, creating secondary porosity. This is evident in the microfacies, where dissolution features and the filling of pore spaces by meteoric sparry calcite cement are visible. Authigenic silica presence as void-filling material suggests silicification postdates micritic matrix dissolution.

Mesogenetic processes affect carbonate rocks during burial, extending from near-surface to metamorphic realms^83^. Burial diagenesis is divided into shallow and deep burial stages. Shallow burial diagenesis occurs during the highstand systems tract (HST), where increasing burial depth leads to mechanical compaction and dolomite cementation. Shallow burial diagenesis is marked by advanced mechanical compaction and dolomite cement development. Distorted fibrous calcite and grain periphery spalling, visible in the microfacies analysis, suggest mechanical compaction followed by marine cementation (Fig. 9g and h)^14,87^. Medium to coarse crystalline dolomite cement (up to 100 mm) precipitated at low to moderate temperatures during shallow burial diagenesis^63,88^. Overgrowth relationships between dolomite and spar cements, observed in microfacies, indicate dolomite cementation postdates dissolution and meteoric cementation [Fig. 10e and f]^89,90^.

Deep burial diagenesis involves fracture formation and burial calcite cementation (fracture-filled sparry calcite cement) (Fig. 10a, b). Deep burial diagenesis is strongly influenced by increasing pressure and temperature associated with continued burial during the later stages of the highstand system tract (HST). Microfacies analysis highlights the role of chemical compaction, likely postdating meteoric cementation^63,91^. Deep burial calcite cement precipitated via pressure dissolution and skeletal particle interpenetration^91^.

Telogenesis is characterized by uplifting diagenesis, wherein carbonate rocks are primarily influenced by meteoric water^83^. Telogenetic processes are associated with emergence and low salinity, oxidizing conditions^66^. Uplifting induces fracture formation and exposure of carbonate strata to meteoric-vadose conditions. Telogenesis is associated with the exposure of strata during regressive phases (RS), where meteoric vadose diagenesis becomes dominant. The microfacies evidence of sparry calcite cement precipitation within some fractures clearly correlates with this final phase of meteoric diagenesis.

## Conclusions

The integration of stratigraphical and petrographical studies on the Lower to Middle Eocene stratigraphic rock units, Minia, Gebel Hof, and Observatory formations, has provided valuable insights into their paleoenvironmental and diagenetic evolution. These rock units are primarily composed of marl, dolomite, and limestone.

The detailed microfacies analysis revealed seven distinct types; bioclastic floatstone (MFT-1), ferruginous dolomite (MFT-2), bioclastic packstone (MFT-3), ferruginous peloidal bioclastic grainstone (MFT-4), sandy peloidal bioclastic rudstone (MFT-5), foraminiferal wackestone (MFT-6), and bioclastic packstone (MFT-7). These microfacies are characterized by a diverse faunal content, including bivalve oyster shells, gastropod shell fragments, serpulid worm tubes, bryozoan fronds, miliolid and larger foraminiferal tests, coral fragments, echinoid plates, planktonic foraminifera, and various algal plates. These microfacies types reflect a variety of depositional environments, ranging from high-energy, shallow subtidal inner ramp to low-energy, open-marine middle ramp settings.

The sequence stratigraphic analysis reveals three depositional sequences. The Minia Formation (YpM-Sq1) records transgressive outer ramp to carbonate-rich highstand deposits, ended by a sequence boundary (SB Yp/Lu) marking a regional sea-level fall. The Gebel Hof Formation (LuGH-Sq2) reflects highstand mid-ramp to lagoonal deposits. The Observatory Formation (LuOb-Sq3) captures renewed transgression and subsequent regression. These sequences correlate with global Ypresian-Lutetian sea-level fluctuations, modulated by Syrian Arc tectonics, which governed accommodation space and facies transitions.

Diagenetic processes have played a significant role in altering the primary features of the studied carbonates, including micritization, glauconitization, cementation, dolomitization, neomorphism, dissolution, silicification, compaction, fracturing, and iron oxides. The diagenetic history is characterized by three distinct episodes; eogenesis, mesogenesis, and telogenesis. The eogenesis episode involves processes such as micritization, glauconitization, marine calcite cementation, early dolomitization, and initial mechanical compaction, reflecting near-surface marine-phreatic and meteoric-phreatic diagenetic conditions. The mesogenesis stage includes late mechanical compaction, dolomite cement precipitation, fracturing, and the formation of burial sparry calcite cement within the burial diagenetic environment. Finally, the telogenesis (uplifting diagenesis) episode involves fracturing, meteoric-vadose cementation (precipitation of fracture-fill sparry calcite cement), and the formation of iron oxides.

The depositional environments exerted a significant influence on the subsequent diagenetic modifications in the studied carbonate rocks. High-energy inner ramp settings, characterized by active water circulation, promoted early cementation and micritization. In contrast, middle ramp environments, which featured slower sedimentation rates, favored dissolution and neomorphism. The restricted conditions at the platform margin created favorable environments for dolomitization and glauconitization. These diagenetic processes, which are intricately linked to the depositional settings, collectively shaped the diagenetic history of the studied successions. By integrating a detailed depositional model, this study provides a comprehensive understanding of the interplay between depositional processes and diagenetic imprints. It offers valuable insights into the paleoenvironmental evolution of carbonate systems in comparable geological settings.

This research highlights the complexity of carbonate diagenesis and emphasizes the necessity of integrating microfacies analysis with diagenetic interpretations to reconstruct paleoenvironmental conditions and diagenetic histories. The findings enhance the understanding of carbonate rock formation processes and establish a framework for interpreting similar carbonate deposits across diverse geological contexts.

## Data Availability

The data used and analysed during the current study is available from the corresponding author on reasonable request.
